# Unilateral biportal endoscopic partial cervical laminectomy and facetectomy: An ex vivo study and case report

**DOI:** 10.1111/vsu.70095

**Published:** 2026-02-27

**Authors:** Hojung Bae, Haebeom Lee, Sanghyun Nam, Youngjin Jeon, Jaemin Jeong

**Affiliations:** ^1^ College of Veterinary Medicine Chungnam National University Daejeon Republic of Korea

## Abstract

**Objective:**

To determine the feasibility of a unilateral biportal endoscopic (UBE) partial cervical laminectomy and facetectomy in canine cervical vertebrae.

**Study design:**

Cadaveric study and case report.

**Sample population:**

Fourteen normal beagle cadavers and a 5‐year‐old spayed female Doberman pinscher.

**Methods:**

Unilateral biportal endoscopic laminectomies were performed at C3–4 and C6–7, extending from the interlaminar junction to the corresponding nerve root, followed by probing. Feasibility was based on the ability to visualize (scored 0–2) and probe anatomical landmarks, complications, and computed tomographic measurements of the bone window. Intraoperative and postoperative parameters were compared between left‐ and right‐sided approaches. One Doberman pinscher with wobbler syndrome underwent UBE laminectomy with vertebral distraction and fusion at C5–6.

**Results:**

Anatomical landmarks were consistently probed, and visualization scores were 2 ± 0. Dural injuries were noted at three sites. Median surgical time was 31.5 (17.5–145) min. Instrument and endoscope portals measured 1.21 ± 0.3 and 1.0 (0.6–1.8) cm, respectively. Laminectomy ratios, defined relative to the contralateral intact lamina, were 39.9% ± 20.6% (cranial) and 20.7% ± 23.9% (caudal). No differences were detected between left‐ and right‐sided approaches. In the clinical case, proprioceptive ataxia resolved within 1 month after surgery, and neurologic examination at the 6 month follow up was normal.

**Conclusion:**

Unilateral biportal endoscopic laminectomy provided adequate visualization in cadaveric specimens and was associated with a successful outcome in one clinical case.

**Clinical significance:**

Unilateral biportal endoscopic laminectomy can be considered a minimally invasive alternative for dorsal decompression of the cervical spine in dogs.

**Abbreviations**
AF: annulus fibrosus
BCS: body condition score
Cd: caudal
CdCrV: caudal margin of cranial vertebra
CdN: caudal region of nerve root
Cr: cranial
CrCdV: cranial margin of caudal vertebra
CrN: cranial region of nerve root
CT: computed tomography
DL: dorsal lamina
F: facet joint
IS: interlaminar space
Lt: left
MRI: magnetic resonance imaging
MISS: minimally invasive spine surgery
NR: nerve root
RF: radiofrequency
Rt: right
SC: spinal cord
UBE: unilateral biportal endoscopic

## 1 INTRODUCTION

Interest in minimally invasive spine surgery (MISS) has increased markedly in veterinary medicine, and research has been conducted with the cervical, thoracolumbar, and lumbosacral regions.^1^, ^2^, ^3^, ^4^ In the cervical spine, techniques such as video‐assisted cervical ventral slot and microendoscope‐assisted cervical dorsal laminectomy have been described.^3^, ^4^ These video‐assisted procedures employ endoscopes to improve visualization of the surgical field and reduce soft tissue disruption compared with conventional open approaches.

Full‐endoscopic MISS has gained increasing attention in human medicine because it can minimize soft tissue damage and provide superior operative visualization.^5^ Full‐endoscopic techniques are classified into uniportal and biportal systems according to the number of working portals. The biportal system offers greater flexibility in instrument manipulation and improved visualization, and has therefore been widely adopted in clinical practice.^6^ Cervical unilateral biportal endoscopy (UBE) has been reported in some human clinical cases, demonstrating feasibility and safety.^7^, ^8^ However, its application in veterinary cervical spine studies has not been described.

Cervical compressive myelopathy is a clinically significant condition in dogs, often caused by intervertebral disc herniation, facet joint hypertrophy, or thickening of the ligamentum flavum. The ventral slot technique remains the most common procedure for ventral or ventrolateral disc herniation. Nevertheless, this approach has limitations due to anatomical constraints when applied to compressive lesions located in the dorsal or lateral aspect of the vertebral canal, and carries a risk of injury to adjacent soft tissues, including the recurrent laryngeal nerve and the esophagus.^9^, ^10^ In such cases, decompression can be achieved by dorsal laminectomy or hemilaminectomy.^11^ However, the dorsal aspects of the cervical vertebrae are covered by substantial musculature, requiring extensive soft‐tissue dissection for surgical access, which may contribute to increased postoperative pain and spinal instability.^4^ These limitations provide a strong rationale for exploring minimally invasive approaches, such as cervical UBE, to address dorsal and lateral compressive lesions more effectively.

The present study was therefore designed to evaluate the feasibility of UBE partial cervical laminectomy and facetectomy via the interlaminar approach in canine cervical vertebrae and to describe the surgical techniques. A clinical case involving the application of cervical UBE laminectomy is also presented to illustrate the clinical applicability of the technique. We hypothesized that percutaneous cervical UBE laminectomy would be technically feasible in dog cadavers without disc extrusion, providing adequate visualization and probing of key anatomical structures within the vertebral canal, such as the spinal cord, nerve root, and intervertebral disc space, while minimizing the risk of injury to vital structures.

## 2 MATERIAL AND METHODS

### 2.1 Cadaveric study

#### 2.1.1 Study subjects

Fourteen beagles were euthanized for reasons unrelated to this study. All cadavers originated from dogs enrolled in unrelated terminal studies, which were approved by the institutional animal care and use committee of Chungnam National University (approval nos.: 202304A‐CNU‐001 and 202404A‐CNU‐006). Body weight and the body condition score (BCS) of each cadaver were recorded. Radiography and computed tomography (CT) (Aquillion Prime SP, TSX‐303B; Canon Medical Systems, Japan) images were produced to confirm the absence of spinal abnormalities, and the length of the interlaminar space at the midline of C3–4 and C6–7 was measured on the CT image. The cadavers were frozen at −20°C and thawed 24 h prior to image examination or the surgical procedures. Preoperative CT images were obtained from the occipital bone to the second thoracic vertebra level of each cadaver, with a slice thickness of 0.5 mm. The cadavers were positioned in sternal recumbency, with the neck slightly flexed to reflect typical surgical positioning. Seven cadavers underwent laminectomy on the left side at C3–4 and on the right side at C6–7, and the remaining seven cadavers underwent laminectomy on the opposite side of each vertebral segment. The sample size was determined with reference to a previously published veterinary MISS study by Julien et al., in which six animals were included per group.^2^ Accounting for an anticipated 10% dropout rate, the sample size in the present study was set at seven cadavers per group. The laminectomy location of each cadaver was assigned using an Excel random number generator (Microsoft, Redmond, Washington, USA).

#### 2.1.2 Surgical procedures

**FIGURE 1:**
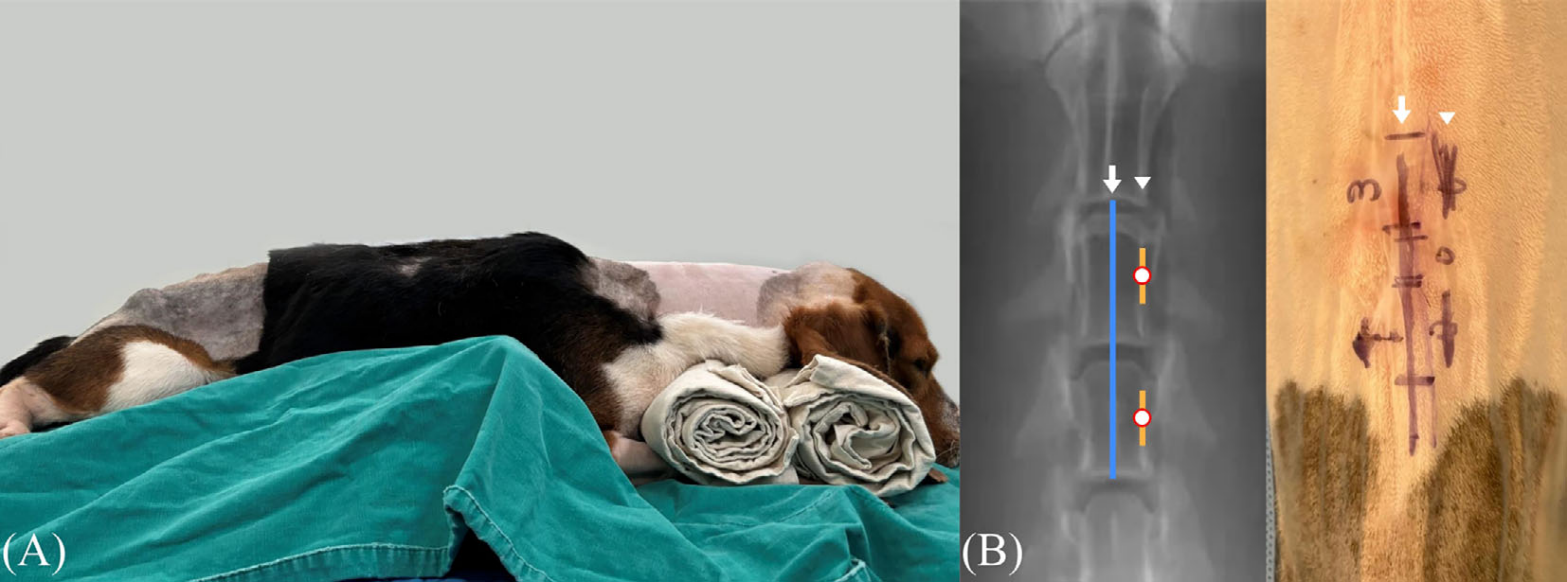
Cadaver positioning (A) and skin incision (B). (A) The cadaver was positioned in sternal recumbency with slight neck flexion supported by a bean bag and rolled towels. (B) Two skin incisions (yellow lines) were made along the medial border of the pedicle (arrowhead). The white arrow indicates the cervical vertebral midline, and the blue line marks the spinous process.

**FIGURE 2:**
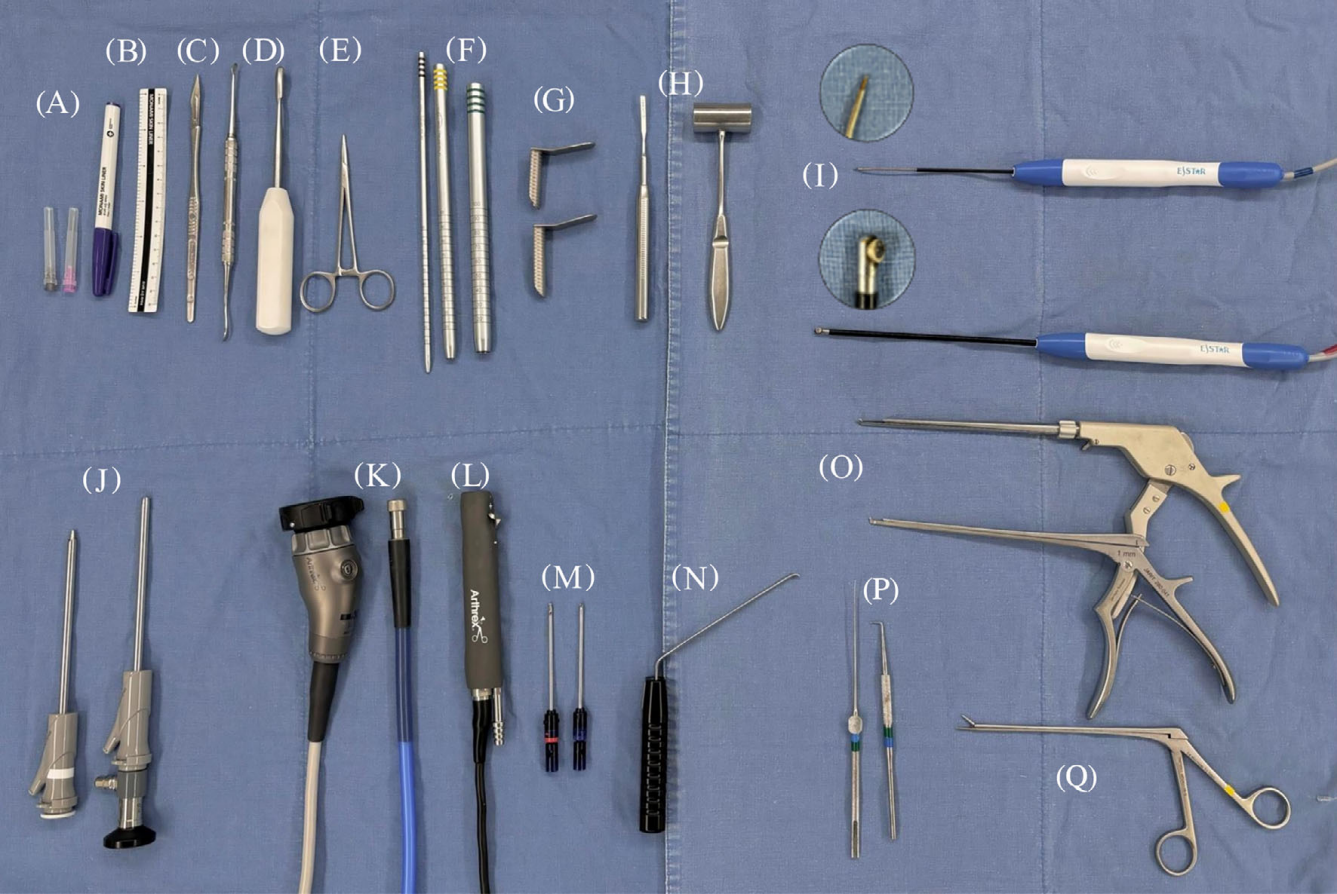
Surgical instruments for unilateral biportal endoscopic (UBE) cervical laminectomy. (A) Needles. (B) Surgical pen and ruler. (C) Scalpel blade. (D) Periosteal elevator. (E) Hemostatic forceps. (F) Serial dilators (5.1, 9.2, 12.7 mm) (Solendos, Seoul, Republic of Korea). (G) Semitubular retractors (Solendos). (H) Chisel and mallet. (I) Radiofrequency (Biounit, Gyeonggi‐do, Republic of Korea). (J) 0° 4 mm endoscope scope (Endovision, Daegu, Republic of Korea). (K) Light cable and camera (Arthrex, Naples, Florida, USA). (L) Saber shaver (Arthrex). (M) Shaver and hooded round burr (Arthrex). (N) Root retractor (Endovision). (O) Kerrison rongeurs with 1‐mm and 2‐mm tip widths (Integra Jarit GmbH, Rietheim‐Weilheim, Germany). (P) Probes. (Q) Pituitary forceps (Solendos).

Prior to the surgical procedure, each cadaver was positioned as above, with its neck slightly flexed to widen the interlaminar space (Figure 1). In particular, the neck was aligned parallel to the surgical table to achieve a straight cervical alignment. The neck flexion angle during surgical positioning was visually measured in the sagittal plane as the angle formed between the longitudinal axis of the neck and a line connecting the head and muzzle. This surgical positioning was applied uniformly regardless of the surgical level. Figure 2 shows the surgical table setting. Hair was clipped from the occiput to the second thoracic vertebra, and an endoscopy spine drape (Endoscopy drape UBE, Sejong Healthcare, Gyonggi‐do, Republic of Korea) was placed over the surgical site.

An 18 gauge needle was directed toward the dorsal lamina and inserted perpendicular to the lamina surface under fluoroscopic guidance to identify the target segment. The landmarks for the portals, located along the medial border of the right or left pedicle and centered on its midpoint, were identified under fluoroscopy guidance (BP Pulsera, Philips Medical Systems, Nederland B.V.). Two cranial‐caudal skin incisions, each approximately 1 cm, were made about 2 cm apart, based on the landmarks identified by fluoroscopy (Figure 1).

Periosteal elevators were inserted through the skin incisions and manipulated with vertical sweeping motions to create a working pathway, after which they were removed. A 9.2 mm serial dilator (Solendos, Seoul, Republic of Korea) was introduced through the skin incision on the surgeon's dominant (right) side, followed by sequential insertion of a 12.7 mm serial dilator to dilate the portal progressively. After removal of the 12.7 mm serial dilator, a 12 mm semitubular retractor (Solendos) was advanced over the 9.2 mm dilator to establish the instrument portal, after which the remaining serial dilator was removed.

**FIGURE 3:**
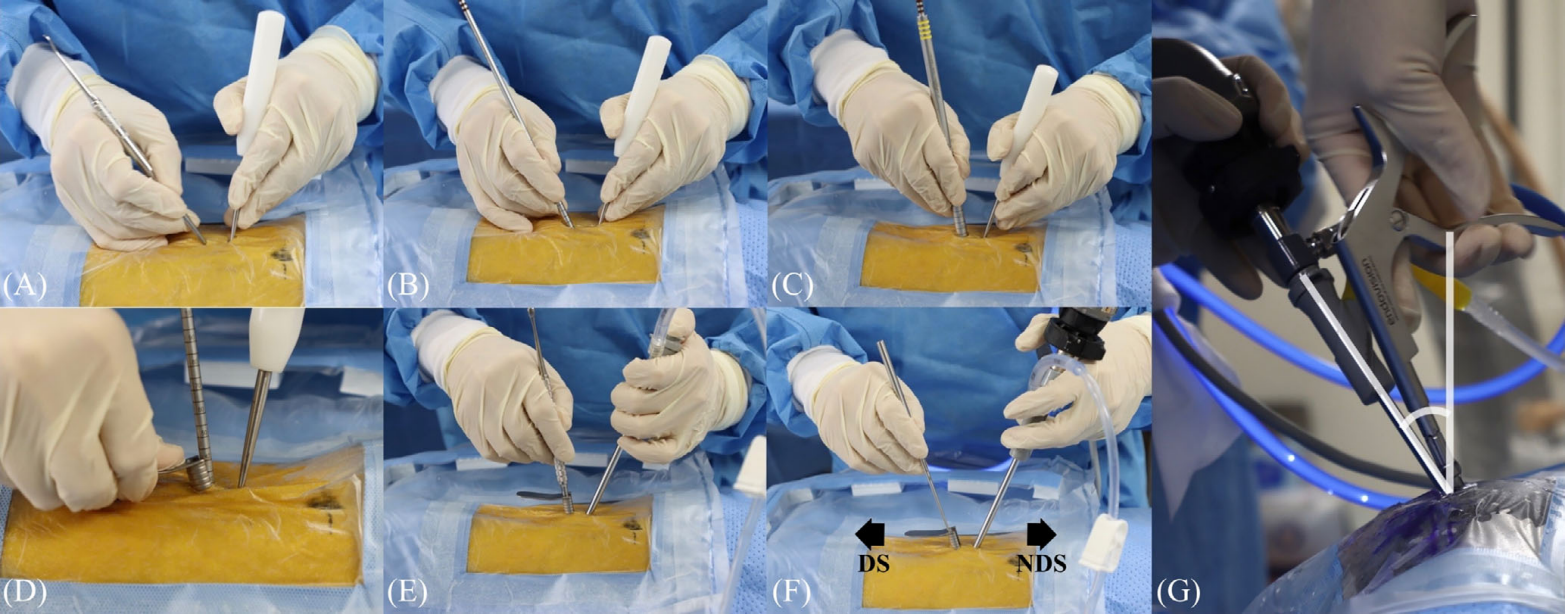
Placement of the scope and instrument portal and measurement of the insertion angle. (A) Periosteal elevators were inserted through the skin incisions and manipulated using vertical sweeping motions to create a working pathway. (B) A 9.2 mm serial dilator was introduced through the skin incision on the surgeon's dominant side. (C) A 12.7 mm serial dilator was subsequently inserted over the 9.2 mm serial dilator to dilate the portal progressively. (D) The 12.7 mm serial dilator was removed and a 12 mm semitubular retractor was advanced over the 9.2 mm dilator to establish the instrument portal. (E) An endoscope sheath with an obturator was inserted through the skin incision located on the surgeon's nondominant side. (F) The obturator was removed and an endoscope connected to a camera was inserted through the sheath. The instrument portal and endoscope portal were created on the dominant side (DS) and nondominant side (NDS) of the surgeon, respectively. (G) The endoscope insertion angle was determined by the angle between the scope and a line perpendicular to the surgical table.

An endoscope sheath with an obturator was then inserted through the skin incision on the surgeon's nondominant (left) side. With the obturator positioned in the endoscope portal and a periosteal elevator placed through the instrument portal, the paraspinal muscles were bluntly elevated using vertical sweeping motions, preserving tendinous attachments. When the initial muscle detachment was sufficient to allow instrument movement without obstruction, the obturator was removed from the sheath. The camera was then connected to the sheath, and continuous irrigation through the endoscope was initiated to maintain a clear surgical field by gravity flow, maintaining hydrostatic pressure at 30 mmHg or lower using a 3 L saline bag suspended 40 cm above the surgical site.^12^ The semitubular retractor maintained the instrument portal and controlled fluid outflow. Surgical instrument was held in the surgeon's (JJ's) dominant (right) hand, and the 0° 4 mm endoscope (Spinuss Scope, Endovision, Daegu, Republic of Korea) was held in the nondominant hand (Figure 3).

**FIGURE 4:**
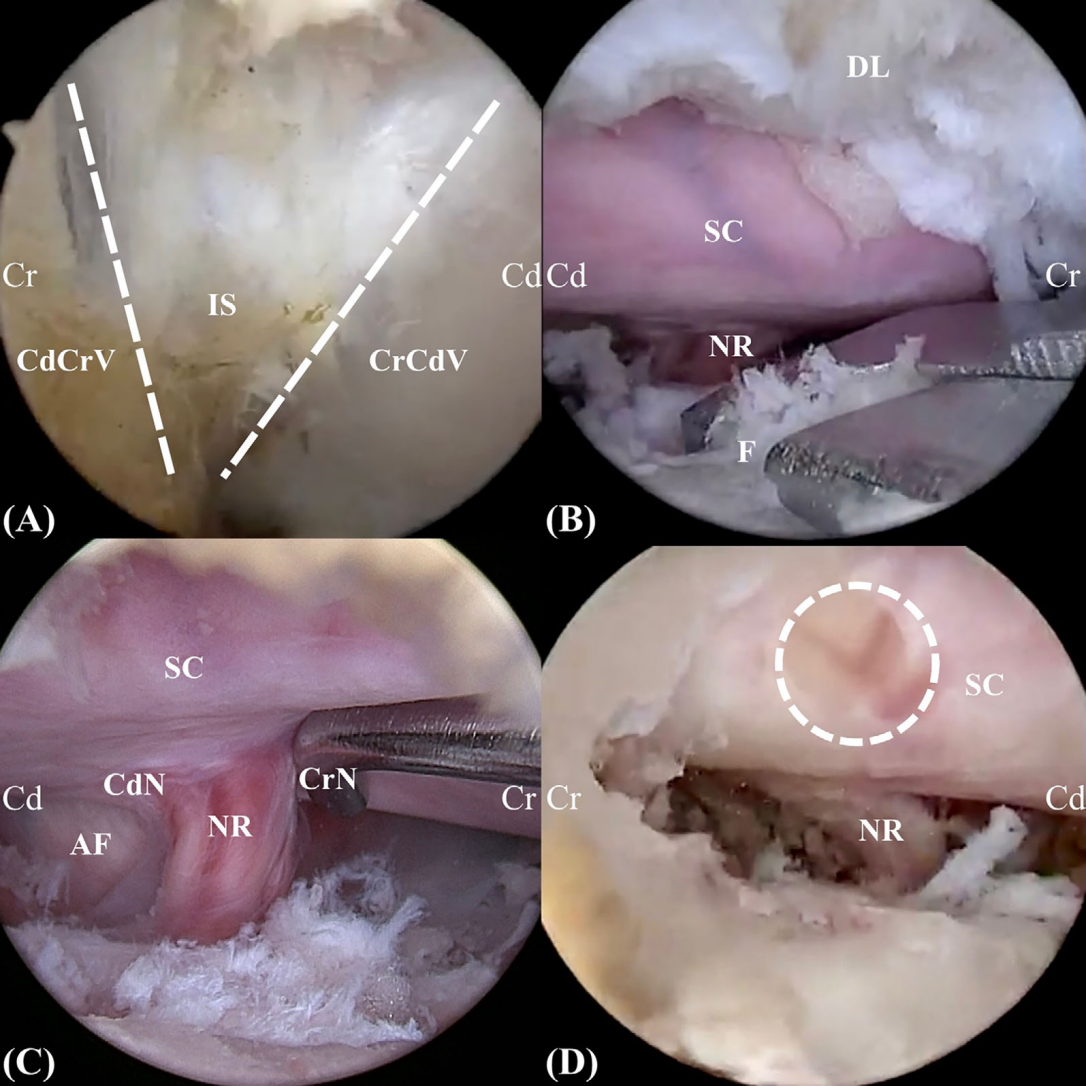
Intraoperative images. (A) Identification of the V point, which refers to the intersection of the cranial margin of the caudal lamina, the caudal margin of the cranial lamina, and the medial aspect of the facet joint. (B) Medial facetectomy performed using a Kerrison rongeur. (C) Nerve root probing. (D) Dural injury (dotted circle) caused during bone removal. Abbreviations: AF, annulus fibrosus; CdCrV, caudal margin of cranial vertebra; CdN, caudal region of nerve root; CrCdV, cranial margin of caudal vertebra; CrN, cranial region of nerve root; DL, dorsal lamina; F, facet joint; IS, interlaminar space; NR, nerve root; SC, spinal cord.

Radiofrequency (RF) (Biounit, Gyeonggi‐do, Republic of Korea) and periosteal elevators were used to detach the epaxial muscles from the bone. For localization and orientation, the anatomical landmark known as the V point was adopted. This was defined as the area where the cranial margin of the caudal lamina, the caudal margin of the cranial lamina, and the medial border of the facet joint converge (Figure 4A).^13^ Starting from the V point, the laminectomy was extended cranially, caudally, and laterally until the nerve root of the corresponding segment was visualized. Partial medial facetectomy was performed if the ventrolateral aspect of the vertebral canal could not be visualized sufficiently. For the limited laminectomy, a high‐speed hooded round burr (Arthrex, Naples, Florida, USA) was initially used to make the bone eggshell thin, and a Kerrison rongeur (Integra Jarit GmbH, Rietheim‐Weilheim, Germany) was subsequently used to remove inner cortical bone and ligamentum flavum (Figure 4B). Bone debris and smoke generated during burring and RF ablation were removed by continuous irrigation. Epidural fat was excised using pituitary forceps as needed. Finally, the cranial and caudal regions of the nerve root and the annulus fibrosus of the intervertebral disc were probed to confirm accessibility of the corresponding structures (Figure 4C). In this study, the ability to probe intracanal structures was used as an indirect measure of the feasibility of removing compressive lesions located at the target region. The intraoperative endoscopy procedure is shown in Supporting Information, Video S1.

#### 2.1.3 Operative and postoperative assessment

Surgical time was recorded for each procedure, and included the duration from the initiation of the procedure to endoscope insertion, and time spent on V point identification, soft tissue dissection (after V point identification), laminectomy and medial facetectomy, probing, and skin closure. The skin incision length and the distance between the two incisions were measured. The endoscope insertion angle during laminectomy was measured on the transverse plane (Figure 3). At the end of the procedure, the surgical field was inspected endoscopically for complications, with particular attention to the integrity of the dural sac, spinal cord, and nerve root.

The visualization score was based on the visibility of major anatomical structures, including the articular facet, V point, ligamentum flavum, spinal cord, cranial and caudal regions of the nerve root, and the intervertebral disc space. A score of 2 (excellent) was assigned when all structures were clearly visualized, 1 (moderate) when more than half were visible, and 0 (poor) when fewer than half were visible (Table 1). The performance assessment score was determined based on the feasibility of laminectomy and probing of the nerve root at the cranial and caudal regions, as well as the annulus fibrosus (Table 1). Scores were rated as 2 (excellent) when all procedures could be performed successfully, 1 (moderate) when more than half were feasible, 0 (poor) when half or fewer could be achieved. Sites achieving a score of 2 in both categories were considered successful. Scores were assessed retrospectively from recorded endoscopic videos by an independent assessor (NS), a surgical resident who was not present in the operating room and was blinded to the lateralization of operative sites.

**TABLE 1:** Visualization and performance scoring system.

Visualization scoring system^a^		Performance scoring system^b^	
Score	Criteria	Score	Criteria
0	Less than four structures visualized	0	Less than two procedures possible
1	Approximately four to six structures visualized	1	Approximately two to three procedures possible
2	All major structures visualized	2	All procedures possible

^a^ Major structures assessed were facet joint, V point, ligamentum flavum, spinal cord, cranial and caudal region of nerve root, and intervertebral disc space.

^b^ Performances assessed for possibility were laminectomy, probing caudal region of the nerve root, probing cranial region of the nerve root, probing annulus fibrosus.

**FIGURE 5:**
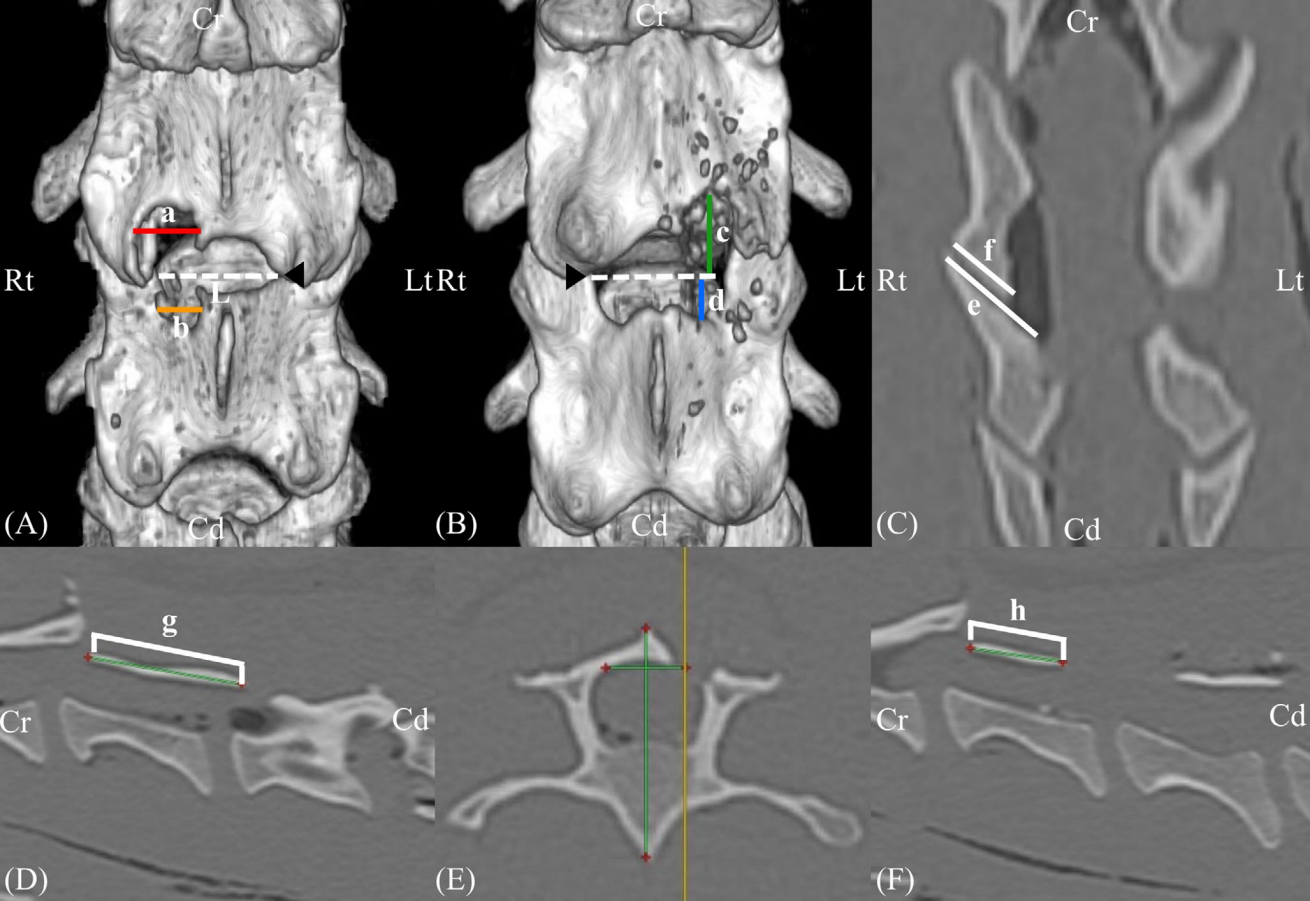
Measurement of the bone window size and the extent of the medial facetectomy. (A) Bone resection rate relative to the facet‐to‐facet length was calculated by dividing the mean width of the maximally resected regions of the cranial and caudal laminae by the facet‐to‐facet length ([a + b/2 L] × 100%) in the figure. Measurements were performed in the frontal view of the three‐dimensional (3D) reconstructed image. (B) Cr:Cd laminectomy ratio was determined as bone window length cranial to the V point (c) divided by bone window length caudal to the V point (d). The bone window length was defined as the distance along the cranial‐caudal axis between the most cranial and the most caudal points of the bone window (represented by c + d in the figure). Both measurements were assessed in the frontal view of a 3D reconstructed image. The black arrowhead indicates the V point. Medial facetectomy was performed on the left facet joint in (B). (C) The extent of the medial facetectomy was calculated as (1 − remaining articular process length of cranial vertebra (f) / intact articular process length of caudal vertebra (e)) × 100 (%) and assessed in the frontal view of the computed tomography (CT) image. (D, F) The laminectomy ratio was determined by (1 − remaining lamina length (h) / intact lamina length (g)) × 100 (%) in the sagittal view of the CT image. Intact lamina length was measured on the contralateral side at a location equidistant from the spinous process to the resected site. Abbreviations: Cd, caudal; Cr, cranial; Lt, left; Rt, right.

Postoperative CT scans were analyzed using a Radiant DICOM viewer 2024.2 (Medixant, Poznan, Poland). Measurements included the bone window length, laminectomy ratio, cranial‐to‐caudal bone window length ratio (Cr: Cd bone window ratio) relative to the V point, the extent of medial facetectomy, and bone resection rate relative to facet‐to‐facet length. Figure 5 shows the measurement methods in detail. The bone window length was measured for subsequent comparison with the standard open cervical approach. The laminectomy ratio normalized the extent of laminectomy to account for individual variations in lamina length, and was used to compare the extent of lamina resection during UBE with the standard open cervical approach. The Cr:Cd bone window ratio was measured to evaluate whether the bone window was centered on the V point and was compared between the left‐ and right‐sided approaches to determine whether differences in the relative cranial‐caudal positioning of the instrument and endoscope portals influenced the pattern of bone resection. The extent of medial facetectomy was assessed with slight modification from a previous study to infer the potential impact of the procedure on spinal stability.^14^ The bone resection rate relative to facet‐to‐facet length was calculated by dividing the mean width of the maximally resected regions of the cranial and caudal laminae by the facet‐to‐facet length, enabling comparison of the resection extent between conventional dorsal laminectomy and the present study.

#### 2.1.4 Statistical analysis

Descriptive data with a normal distribution were presented as means ± SDs, and non‐normally distributed data were presented as medians (ranges). Statistical analyses were conducted using SPSS software version 30 (IBM Corporation, Armonk, New York, USA). Differences in visualization scores, performance scores, total surgical times, skin incision lengths and intervals, endoscope insertion angles, bone window dimensions, and the laminectomy ratio were compared between left‐ and right‐sided approaches. Normality was assessed using the Shapiro–Wilk test. Non‐normally distributed data were evaluated using the Mann–Whitney test to determine statistical significance. A *p*‐value of less than .05 was considered statistically significant. Total surgical time was analyzed using cumulative sum (CUSUM) and polynomial regression to identify the learning curve.

### 2.2 Clinical case report

**FIGURE 6:**
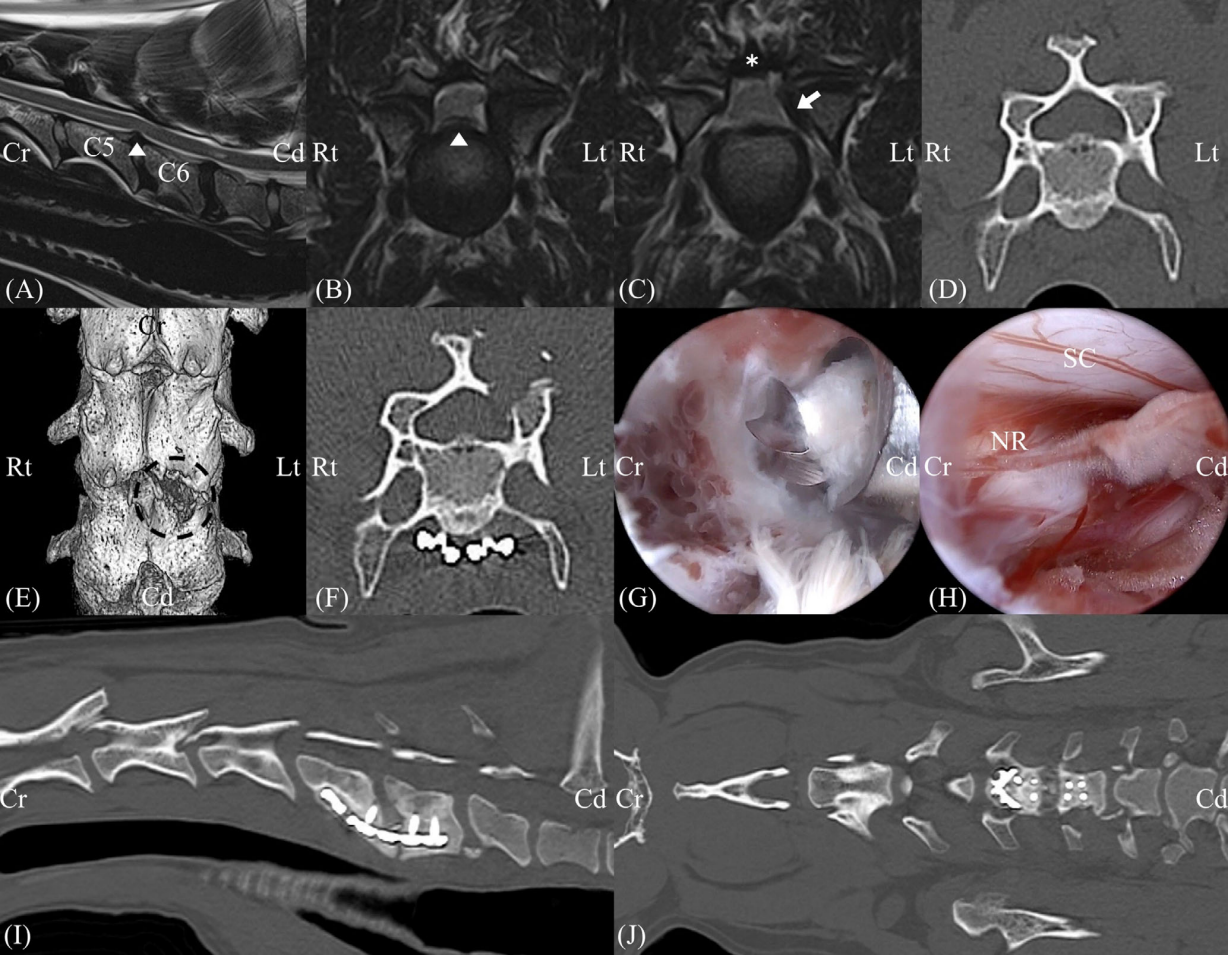
Preoperative, intraoperative, and postoperative images of a 5‐year‐old Doberman pinscher with cervical myelopathy. (A, B) Preoperative magnetic resonance imaging (MRI) showing disc protrusion (arrowhead) at C5‐6 with subsequent spinal cord compression. (A: T2W1, sagittal plane; B: T2W1, transverse plane). (C) Preoperative MRI image demonstrating a compressive lesion (arrow) medial to the left articular facet of the cranial C6. Asterisk indicates ligamentum flavum thickening (T2W1, transverse plane). (D) Preoperative computed tomography (CT) image showing vertebral canal dorsoventrally flattened (bone window, transverse plane). (E) Postoperative three‐dimensional (3D) reconstructed CT image with laminectomy and medial facetectomy (black circle). (F) Postoperative CT image (bone window, transverse plane). (G) Laminectomy performed using a hooded round burr. (H) Spinal cord and nerve root exposed after laminectomy and partial medial facetectomy. (I) Postoperative CT image (bone window, sagittal plane) showing ventral bony bridge formed 6 months postoperatively. (J) Postoperative CT image (bone window, horizontal plane). Abbreviations: Cd, caudal; Cr, cranial; Lt, left; NR, nerve root; Rt, right; SC, spinal cord.

A 5‐year‐old spayed female Doberman pinscher, weighing 33.3 kg, with proprioceptive ataxia characterized by a so‐called two‐engine gait, was presented to Chungnam Veterinary Teaching Hospital. Neurological examination revealed cervical pain upon palpation and delayed postural reactions in the pelvic limbs. Transverse CT images (bone window) of C4–C7 showed a dorsoventrally flattened, rectangular vertebral canal, resulting in a trapezoidal spinal cord. Magnetic resonance imaging (MRI) demonstrated ventral intervertebral disc protrusion at C5–6, C6–7, and C7–T1, with definitive spinal cord compression only at C5–6. Ligamentum flavum thickening and hypertrophy of the left medial articular facet were also observed at C5–6 (Figure 6). Based on imaging and clinical findings, the patient was diagnosed with osseous‐ and disc‐associated cervical spondylomyelopathy, with C5–6 spinal cord compression considered the primary contributor to clinical signs.

Cervical UBE laminectomy and medial facetectomy were planned on the left side of the C5–6 segment to achieve dorsolateral spinal cord decompression. Preoperative CT images indicated that resection of approximately 20% of the left C5 lamina and 30% of the left C6 lamina would relieve dorsal and dorsolateral compression. Ventral interbody distraction and fusion were also planned to address the ventral disc herniation and stabilize the vertebral segment. Preoperative coagulation testing confirmed type 1 von Willebrand disease, and desmopressin acetate (0.1 μg/kg once daily) was administered perioperatively.

The patient was positioned in sternal recumbency with slight neck flexion. Two skin incisions were made along the medial border of the left pedicle, approximately 2 cm apart. After blunt muscle elevation through the portal using the dilator and scope sheath with obturator, the endoscope camera was inserted after removal of the obturator. After identification of the V point, dorsal laminectomy and partial medial facetectomy were performed, and thickened ligamentum flavum was resected (Figure 6; Supporting Information, Video S2). Intraoperative hemorrhage was controlled using ball‐tip RF, absorbable bone hemostatic agent (Novoseal, CG Bio, Seoul, Republic of Korea), and gelatin‐thrombin matrix (Floseal, Baxter, Illinois, USA).

Following completion of the cervical UBE laminectomy, the patient was repositioned in dorsal recumbency for the ventral procedure. An anterior cervical distractor (ACIF distractor, Jeil Medical, Seoul, Republic of Korea) was utilized for C5–6 intervertebral space distraction. A 4 × 11.5 × 12.5 mm cage (Jeil Medical) packed with autogenous cancellous bone graft was placed between the vertebral bodies of C5 and C6 and impacted. Two 2.4 mm six‐hole locking plates (Jeil Medical) were applied ventrally across C5 and C6. A closed suction drain was placed at the ventral surgical site.

## 3 RESULTS

### 3.1 Cadaveric study

#### 3.1.1 Ex vivo specimens

The 14 cadavers had a mean body weight of 9.98 ± 2.1 kg and a median body condition score (BCS) of 5.5 (range 2–7) on a 9‐point scale.

#### 3.1.2 Intraoperative records

**FIGURE 7:**
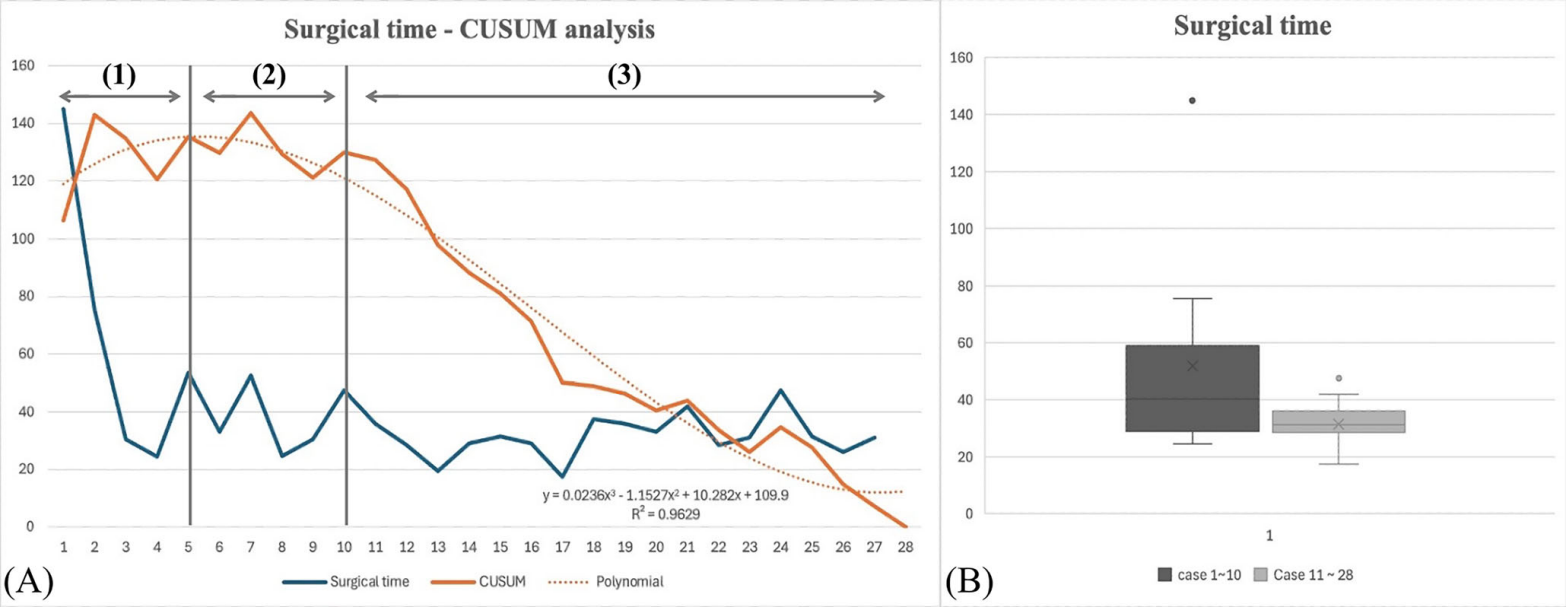
Cumulative sum (CUSUM) analysis of the learning curve for 28 cadaveric procedures. (A) The CUSUM curve shows an upward trend in the first two cases (phase 1), a plateau from cases 3 to 10 indicating a transitional phase (phase 2), and a downward slope from case 11, reflecting progression to phase 3, where procedural proficiency is achieved. (B) Mean surgical times before and after case 11 were 51.71 ± 36.54 and 31.47 ± 7.06 min, respectively.

The median total surgical time was 31.5 (17.5–145) min. The median time from the start of surgery to scope insertion was 3 (1–20) min. Identification of the V point required a median of 4.25 (1.5–61) min. Soft tissue dissection after V point identification had a median duration of 4.5 (2–15) min. Median times for laminectomy and probing of cranial and caudal regions of the nerve root and annulus fibrosus were 15.25 (3–58) min and 1 (0.5–15) min, respectively. Skin closure required a median of 1.5 (1.0–2.5) min (Table 2). The soft tissue dissection time was longer at C3–4 than at C6–7 (*p* = 0.044), whereas the other components of surgical time did not differ between the two segments (*p* > 0.05). Based on the total operative times from the first to the 28th procedures, a CUSUM graph was generated and represented as a polynomial graph (Figure 7).^15^ In the graph, the first five sites showed the inclination of the CUSUM curve, representing the initial learning phase. The plateau from the sixth to the 10th sites corresponds to phase 2, the transition phase. From the 11th site, the CUSUM curve declined, indicating that the surgeon reached phase 3. The mean surgical time before and after the 11th site was 51.71 ± 36.54 min and 31.47 ± 7.06 min, respectively (*p* = .16).

**TABLE 2:** Surgical time records.

Surgical time records						
	From initiation to endoscope insertion	V point identification	Soft tissue dissection	Laminectomy and medial facetectomy	Probing^a^	Skin closure
Median (range) (min)	3 (1–20)	4.25 (1.5–61)	4.5 (2–15)	15.25 (3–58)	1 (0.5–15)	1.5 (1–2.5)

^a^ Probed structures included cranial and caudal regions of the nerve root and annulus fibrosus.

The mean neck flexion angle was 131.5 ± 2.82°. The mean skin incision length of the instrument portal was 1.21 ± 0.31 cm, and the median skin incision length of the endoscope portal was 1.0 (0.6–1.8) cm. The distance between the two portals was 1.44 ± 0.32 on average. The average scope insertion angle was 18.58 ± 6.70° lateral from the perpendicular on the transverse plane.

Partial medial facetectomy was performed in 8/28 cases to further expand the surgical corridor and improve visualization of the deep‐seated structures. The visualization and performance assessments were given scores of 2 in all sites, indicating the success of the procedure. Comparisons were made between the left approach group and the right approach group, and detailed information is presented in Table 3 and Supporting Information, Table S1.

**TABLE 3:** Representative values of intraoperative record and bone window dimensions, and statistical significance of differences between the left and right approaches.

		*p*‐value^b^	
	Representative value^a^	C3‐4	C6‐7
Surgery time	31.5 (17.5–145) min	.90	.62
Incision length (instrument portal)	1.21 ± 0.31 cm	.32	.9
Incision length (endoscope portal)	1.0 (0.6–1.8) cm	.54	.38
Incision interval	1.44 ± 0.32 cm	.13	.21
Insertion angle	18.58 ± 6.70°	.54	1
Bone window length	14.03 ± 2.42 mm	.9	.53
Laminectomy ratio (Cr)	31% (16% to 90%)	1	.21
Laminectomy ratio (Cd)	20% (0% to 100%)	.38	1
Bone window Cr:Cd ratio	1.58 ± 0.73	.9	.26

Abbreviation: Cd, caudal; Cr, cranial.

^a^ Representative values are presented as means ± SDs for normally distributed data and medians (ranges) for non‐normally distributed data.

^b^ The *p*‐value for the comparison between left and right approaches for C3‐4 and C6‐7.

#### 3.1.3 Intraoperative complication

Intraoperative complications occurred in three sites (10.7%), all of which were related to dural injuries (Figure 4D). The first complication occurred during the probing phase when the endoscope was brought too close to the dura, causing contact and resulting in dural damage. Immediately following this contact, we observed a wrinkling/creasing of the dura at the point of interaction with the endoscope. The second one occurred during laminectomy, while using a round burr. The third one was caused by needling during target segment identification. The three cases of dural tear occurred at the second, eighth, and ninth sites, respectively.

#### 3.1.4 Computed tomographic scan finding

Preoperative CT analysis revealed that the mean interlaminar space length of C6–7 was 1.49 ± 0.89 times longer than that of C3–4. Postoperative CT images were analyzed to measure bone window dimensions and the extent of medial facetectomy. The median laminectomy ratios for cranial vertebrae were 31% (16% to 90%) and for caudal vertebrae were 20% (0%–100%), and the total laminectomy length averaged 14.03 ± 2.42 mm. The ratio of the bone window length cranial and caudal to the V point averaged 1.58 ± 0.73. The average bone resection rate relative to facet‐to‐facet length was 31.29 ± 8.86. Of 28 sites, medial facetectomy was performed in eight sites, seven of which involved C6–7; only one was performed at C3–4. Among the subjects that underwent medial facetectomy, the extent of the facetectomy averaged 31.88% ± 6.08%.

### 3.2 Clinical case outcome

The incision length of the instrument and scope portals were 1.0 and 0.8 cm, respectively. Bone window dimensions were evaluated on immediate postoperative CT images. The total bone window length was 1.43 cm, and laminectomy ratios of C5 and C6 were 13% and 26%, respectively. The extent of facetectomy was 32%. The patient recovered without major complications, except for transiently delayed postural reactions of both thoracic limbs, which resolved 6 days after surgery. Marked improvement in the two‐engine gait was observed 1 week postoperatively, and the patient was discharged 2 weeks after surgery. By 1 month after surgery, the patient exhibited complete resolution of proprioceptive ataxia. Supporting Information, Video S3 shows the patient's perioperative gait. At 6 months postoperatively, CT revealed formation of a ventral bony bridge, and neurological examination showed normal responses in all assessed parameters, including proprioception. Midterm telephone follow up at 8 months postoperatively confirmed that the dog was living without any clinical signs, and owner satisfaction was high.^16^

## 4 DISCUSSION

This study evaluated the feasibility of cervical UBE laminectomy using an interlaminar approach in dogs. In the cadaveric study, the procedure was conducted at C3–4 and C6–7 in 14 cadavers. Excellent visualization of the surgical field was achieved at all sites, and laminectomy, with probing of the nerve roots and disc space, was completed successfully. Vital structures were preserved in 25 of 28 sites, with intraoperative dural injury occurring at three sites.

In the clinical case, cervical UBE laminectomy and medial partial facetectomy, combined with ventral distraction and fusion, were performed at C5–6 in a dog diagnosed with wobbler syndrome. The patient recovered without major complications, and the characteristic two‐engine gait improved gradually postoperatively. These results support the hypothesis that percutaneous cervical UBE laminectomy is technically feasible in dog cadavers without disc extrusion and also demonstrate its potential applicability in a live patient.

Establishing an optimal entry point and trajectory is essential for adequate visualization and to minimize intraoperative complications in endoscopic spine surgery.^13^ Lateral placement of the entry point increases the risk of vertebral artery injury and requires more extensive medial facetectomy to visualize the vertebral canal. Medial placement produces a steep endoscopic trajectory, limiting visualization and probing of intracanal structures. The standard entry point in human cervical UBE is typically located over the medial border of the pedicle. In the present study, skin incisions were created using this landmark. The endoscope was inserted at approximately 18° lateral to a line perpendicular to the surgical table, directed toward the V point. This approach provided adequate visualization of the surgical site and facilitated laminectomy and probing in the canine cervical vertebrae.

Accurate localization and orientation are fundamental in MISS, as successful achievement of the surgical objective through small incisions depends on precise targeting of the pathological site and establishment of an appropriate trajectory. Anatomical landmarks are essential for confirming both location and trajectory.

In human cervical MISS, the V point serves as an important anatomical landmark.^13^ A corresponding anatomical landmark was identified during the cervical interlaminar approach in the cadaveric models, and is applicable to canine UBE laminectomy. However, the location of the V point may vary with the degree of neck flexion. Positioning the neck slightly flexed facilitates visualization of the interlaminar space and prevents medialization of the V point, avoiding excessive bone removal.^13^

There is no consensus regarding optimal portal positioning in human cervical UBE. Portal placement is generally determined by the surgeon's handedness, with the instrument portal on the dominant side and the endoscope portal on the nondominant side.^13^ In some cases, however, the endoscope portal is positioned cranially and the instrument portal caudally, irrespective of handedness, to minimize bone excision.^6^

In the present study, the endoscope portal was consistently placed on the surgeon's nondominant side and the instrument portal on the dominant side. As a result, the craniocaudal orientation of the portals varied depending on whether the procedure was performed on the left or right side of the specimens. No differences were observed between left‐ and right‐sided approaches in incision length and interval, endoscope insertion angle, surgical time, bone window dimension, or procedural feasibility. These findings indicate that using the dominant hand for instrument manipulation and the nondominant hand for endoscope control is an appropriate strategy for canine cervical UBE.

In the present study, laminectomy alone did not provide sufficient visualization at some sites to achieve the intended surgical objective. When the ventral disc space could not be adequately visualized or successfully probed, additional medial partial facetectomy was performed to expand the working corridor in 8 of 28 cases. This requirement occurred predominantly at C6–7, where 88% of the medial partial facetectomies were conducted. This tendency likely reflects the closer proximity of the facet joint to the V point at this level compared with C3–4, and the presence of cervical intumescence in the C6–7 segment.^17^

A major concern associated with medial partial facetectomy is the potential for segmental spinal instability.^18^ A previous cadaveric study demonstrated that unilateral and bilateral facetectomy increased the range of motion in flexion, extension, and axial rotation significantly in dogs.^18^ That study, however, was conducted ex vivo after removal of the paraspinal musculature, and the effect of partial facetectomy on spinal stability was not investigated. Under in vivo conditions, spinal stability may also be influenced by factors such as the integrity of paraspinal muscles, intervertebral disc, and ligamentous structures.

Evidence from human spine surgery suggests that cervical partial facetectomy involving less than 50% of the facet joint does not result in spinal instability.^19^ In the present study, the mean extent of medial facetectomy was 32.8%, and it remained less than 50% in all sites.

Given the limited extent of facetectomy and the minimally invasive nature of the UBE technique, partial excision of the facet joint may be performed with laminectomy to improve visualization and probing of the surgical sites without substantially compromising spinal stability. Nevertheless, further research is warranted to evaluate the impact of endoscopic partial facetectomy on spinal stability in canine cervical vertebrae.

Traditional open cervical dorsal decompression surgery in dogs has been associated with a high rate of postoperative complications, including neurologic deterioration and spinal instability.^20^ This high complication rate, ranging from 25% to 70%, has been attributed to the invasiveness of the dorsal cervical approach, during which paraspinal muscles covering the dorsal aspect of the cervical vertebrae are extensively dissected and ligamentous attachments are disrupted.^11^, ^20^, ^21^ In contrast, the UBE technique minimizes soft tissue disruption, as demonstrated by the small skin incisions measuring approximately 1 cm in the present study. Preservation of the paraspinal muscles and their tendinous attachments further distinguishes this technique from the conventional open approach, with potential benefits for postoperative paraspinal stability.^22^

Extensive bony resection also contributes to postoperative morbidity.^23^ In conventional cervical dorsal laminectomy or hemilaminectomy, bone resection typically involves complete removal of the lamina between both facet joints or unilateral removal of the lamina together with the facet joint and pedicle.^11^, ^24^

In human posterior cervical endoscopic decompression, the cranial and caudal limits of the bone window are typically defined by the insertion and origin of the ligamentum flavum, and the lateral margin is generally restricted to the cranial tip of the superior articular process.^13^ In the present study, resection involved 31.29% of the lamina relative to the facet‐to‐facet width. In terms of laminar length, a median of 31% of the cranial lamina and 20% of the caudal lamina was removed. This extent of bony resection is comparable to the bone window typically created in endoscopic posterior cervical decompressive procedures reported in the human literature. Although direct evaluation of decompressive efficacy was not available in this study, this limited extent of bone resection was sufficient to allow clear visualization and probing of the nerve roots and annulus fibrosus. These findings suggest that the proposed approach may provide adequate access for the removal of compressive lesions within this region, while minimizing the bony resection required. Given its minimally invasive nature, UBE may represent an alternative to traditional open cervical dorsal decompression surgery, possibly maintaining greater postoperative spinal stability.

Compared with uniportal full endoscopic MISS, UBE offers an increased degree of freedom for instrument manipulation because endoscope and working instruments are operated through two separate portals.^6^ The primary instruments employed in UBE are also compatible with those used in arthroscopy, allowing surgeons with an arthroscopic background to adapt to instrument manipulation and improve their proficiency more quickly.^25^ The surgeon in this study had 8 years of experience in arthroscopy. Learning curve analysis based on surgical time revealed that the surgeon underwent an initial learning phase through the first five procedures. The surgeon gained additional experience between the sixth and tenth sites, ultimately achieving expert competence by the 11th site. Mean surgical time before and after achieving expert competence was 51.7 and 36.5 min, respectively, with no intraoperative complications observed after the 10th site. The time spent on soft tissue dissection was longer at C3–4 than at C6–7 (*p* = 0.044), whereas the other components of surgical time did not differ between the two segments (*p* > 0.05).

Irrigation fluid plays an important role in maintaining a clear surgical view and achieving hemostasis in MISS. In comparison with uniportal MISS techniques, UBE has the advantage of facilitating continuous drainage of irrigation fluid through a working portal distinct from the scope portal.^6^ This prevents the stagnation of irrigation fluid and provides a clear surgical view. Debris and smoke produced during burring and RF application are also cleared through continuous irrigation.

In the present study, dural injury (3/28) was the only intraoperative complication. One injury occurred due to contact between the camera and the dural sac, another during laminectomy with a round burr, and the third during needling for fluoroscopic localization of the target segment. Dural injury is the most common complication in endoscopic spine surgery in human medicine. The misperception of the visual field under an endoscopic two‐dimensional plane and poor visualization are considered the main causes.^26^ Familiarity with the endoscopic view during the learning curve and careful instrument handling are essential to reduce this risk.

All dural tears in this study occurred at the second, eighth, and ninth surgical sites, before the surgeon achieved expert proficiency, suggesting an association with the learning curve. Technical refinements may also reduce risk. Thinning the inner cortical bone layer to an eggshell thickness during initial burring and then removing the bony shell with a Kerrison rongeur helps reduce the risk of dural injury during laminectomy.^27^ Although needle localization can shorten operative time, insertion through the interlaminar space may cause dural or spinal cord injury. Careful lateral needling under fluoroscopic guidance or localization using a small serial dilator to palpate the lamina is therefore recommended as a safer alternative.^13^, ^28^ As the caudal cervical vertebrae have a wider interlaminar space than cranial cervical vertebrae, the surgeon should also be more cautious when approaching the caudal cervical area to avoid iatrogenic damage to the dural sac.

Some differences were noted between the clinical case and the cadaveric study. First, the cadaveric procedures were performed only to assess feasibility, so the laminectomy was limited to the extent required to probe the ventrolateral aspect of the vertebral canal. In contrast, the clinical case involved laminar morphological abnormalities causing spinal cord compression, and bone removal was tailored to achieve adequate decompression by excising the affected laminar region.

Second, unlike the cadaveric specimens, the clinical procedure involved active bleeding, which likely contributed to a longer operative time. Minor hemorrhage was controlled with irrigation, whereas more substantial bleeding required adjunctive hemostatic techniques, including Novoseal (CG Bio), and Floseal (Baxter).^29^

In human medicine, where cervical UBE has been studied extensively, clear indications and contraindications have been established.^30^, ^31^ Reported contraindications include severe central stenosis, spinal instability, spinal tumor, and high‐grade spinal deformities such as scoliosis. However, given the limited clinical experience in dogs, indications and contraindications for cervical UBE in veterinary medicine cannot be defined solely on the basis of the present findings. Successful probing of the annulus fibrosus and nerve root regions indirectly supports the feasibility of removing compressive lesions in these areas. Whether adequate decompression can be achieved for lesions located in other regions using cervical UBE laminectomy requires further investigation. In chronic cases with dense adhesions between herniated disc material and the dura, the procedure may be contraindicated or may require intraoperative conversion to an open approach.

Sliding UBE techniques using additional portals for multilevel disease, as well as contralateral decompression through a unilateral approach, have been reported in human spine surgery. These modifications were beyond the scope of the present study.^13^ Accumulation of clinical experience is necessary to refine case selection criteria and define the clinical limitations of this technique in veterinary practice.

Several limitations should be acknowledged. First, assessment of surgical time may have been affected by the initial learning curve. Second, as an ex vivo study, complications such as bleeding, epidural hematoma, or postoperative neurological deterioration could not be evaluated, and intraoperative complications may therefore have been underestimated.

As this study was performed using normal beagle cadavers without intervertebral disc disease, the decompressive efficacy of this technique could not be assessed. The absence of a comparison group also precluded direct comparison between cervical UBE laminectomy and conventional cervical dorsal laminectomy in terms of complication rate and bone window size. Finally, the present study evaluated cervical UBE laminectomy in C3–4 and C6–7; the feasibility and technical aspects in other cervical vertebrae segments remain unknown. The direct impact of the surgical procedure on spinal stability was also not evaluated.

The study demonstrated the technical feasibility of canine cervical UBE laminectomy with an interlaminar approach in a cadaveric model and a single clinical case. The surgical technique provided sufficient visualization and allowed adequate probing of the surgical site. The cadaveric study could not evaluate the removal of extruded disc material or prognosis but its successful application in a clinical patient suggests that the cervical UBE technique can be used as an alternative to conventional open spine surgery, providing minimized postoperative instability in clinical patients with disc extrusion. Further study is needed in clinical cases with disc extrusion to validate the clinical applicability of UBE in canine cervical compressive myelopathy and to compare postoperative pain assessments and other potential advantages compared with traditional open approaches.

## CONFLICT OF INTEREST

The authors declare no conflict of interest related to this study.

## Supporting information


**Table S1.**Surgical time, incision length and interval, insertion angle, and bone window dimension based on the location of laminectomy

**Video S1.** Intraoperative endoscopic view of cadaveric study. This material is available as part of the online article from: link. This supplementary video is edited using VLLO version 13.6.0 (Vismosoft, Seoul, Republic of Korea).

**Video S2.** Intraoperative endoscopic view of the clinical case. This material is available as part of the online article from: link. This supplementary video is edited using VLLO version 13.6.0 (Vismosoft).

**Video S3.** Perioperative gait of a clinical case. This material is available as part of the online article from: link. This supplementary video is edited using VLLO version 13.6.0 (Vismosoft).
