# Supplementary material for: Unilateral biportal endoscopic partial cervical laminectomy and facetectomy: An ex vivo study and case report
Source: Vet Surg. 2026 Feb 27;55(3):657–71. doi: 10.1111/vsu.70095 (PMC13069243; doi:10.1111/vsu.70095)
Supplement: Supplementary file 1 — Table S1.Surgical time, incision length and interval, insertion angle, and bone window dimension based on the location of laminectomy [file VSU-55-657-s004.docx]

**Table S1:** Surgical time, incision length and interval, insertion angle, and bone window dimension based on the location of laminotomy

| Site | Side | Surgery time  (minutes) | Incision length  (IP) (cm) | Incision length  (EP) (cm) | Incision interval  (cm) | Insertion angle (°) | Bone window length (mm) | Laminotomy ratio  (Cr) (mm) | Laminotomy ratio  (Cd) (mm) | Bone window  Cr : Cd ratio |
| --- | --- | --- | --- | --- | --- | --- | --- | --- | --- | --- |
| C3-4 | Lt | 42  (28.5-53.5) | 1.2  (1-2) | 1.0  (0.8-1.8) | 1.4  (0.9-1.6) | 20  (6-24) | 12.04  (10.65-15.12) | 28  (16-35) | 18  (3-35) | 1.92  (1.6-2.81) |
|  | Rt | 36  (30.5-145) | 1.2  (0.9-1.3) | 1.0  (0.6-1.2) | 1.5  (1.3-2.0) | 22  (7-32) | 13.02  (11.46-14.25) | 26  (17-46) | 18  (0-23) | 1.86  (1.02-3.4) |
| *P-*value | | 0.90 | 0.32 | 0.54 | 0.128 | 0.54 | 0.9 | 1 | 0.38 | 0.9 |
| C6-7 | Lt | 26  (17.5-75.5) | 1.1  (0.6-1.9) | 1.0  (0.7-1.4) | 1.5  (1.2-1.9) | 16  (14-24) | 17.38 (12.5-18.63) | 72  (20-90) | 31  (0-100) | 1.4  (0.38-2.02) |
|  | Rt | 30.5  (19.5-36) | 1.1  (0.9-1.5) | 1.0  (0.9-1.3) | 1.3  (0.5-1.7) | 18  (6-30) | 14.51 (11-17.6) | 45  (25-72) | 22  (0-75) | 0.88  (0.23-1.85) |
| *P-*value | | 0.62 | 0.9 | 0.38 | 0.21 | 1 | 0.53 | 0.21 | 1 | 0.26 |

a : p between C3-4 Lt and Rt, b : p between C6-7 Lt and Rt
(Abbreviations : Cd, caudal; Cr, cranial; IP, Instrument portal; EP, Endoscope portal)
